# Stimuli‐Responsive Antibacterial Materials: Molecular Structures, Design Principles, and Biomedical Applications

**DOI:** 10.1002/advs.202104843

**Published:** 2022-02-27

**Authors:** Xianghong Wang, Mengyao Shan, Shike Zhang, Xin Chen, Wentao Liu, Jinzhou Chen, Xuying Liu

**Affiliations:** ^1^ School of Materials Science and Engineering The Key Laboratory of Material Processing and Mold of Ministry of Education Henan Key Laboratory of Advanced Nylon Materials and Application Zhengzhou University Zhengzhou 450001 China; ^2^ College of Food Science and Engineering National Engineering Research Center for Wheat & Corn Further Processing Henan University of Technology Zhengzhou 450001 China

**Keywords:** antibacterial theranostics, bacterial metabolites stimuli, molecular structures, physical stimuli, stimuli‐responsive materials

## Abstract

Infections are regarded as the most severe complication associated with human health, which are urgent to be solved. Stimuli‐responsive materials are appealing therapeutic platforms for antibacterial treatments, which provide great potential for accurate theranostics. In this review, the advantages, the response mechanisms, and the key design principles of stimuli‐responsive antibacterial materials are highlighted. The biomedical applications, the current challenges, and future directions of stimuli‐responsive antibacterial materials are also discussed. First, the categories of stimuli‐responsive antibacterial materials are comprehensively itemized based on different sources of stimuli, including external physical environmental stimuli (e.g., temperature, light, electricity, salt, etc.) and bacterial metabolites stimuli (e.g., acid, enzyme, redox, etc.). Second, structural characteristics, design principles, and biomedical applications of the responsive materials are discussed, and the underlying interrelationships are revealed. The molecular structures and design principles are closely related to the sources of stimuli. Finally, the challenging issues of stimuli‐responsive materials are proposed. This review will provide scientific guidance to promote the clinical applications of stimuli‐responsive antibacterial materials.

## Graphical abstract

This review systematically summarizes the advantages, the response mechanisms, and the key design principles of stimuli‐responsive antibacterial materials. The biomedical applications, the current challenges, and development trends of stimuli‐responsive antibacterial materials are also discussed. Stimuli‐responsive antibacterial materials are appealing to achieve intelligent and personalized medicine, that can avoid the formation of biofilm and inhibit the emergence of drug‐resistant bacteria.

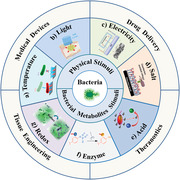

## 1 Introduction

Infections, especially hospital‐acquired infections, present a worldwide healthcare risk and cause severe casualties every year. Similar to the COVID‐19 pandemic, the spread of drug‐resistance endangers public health. If left unchecked, by 2050, bacterial infections might cause the death of about 10 million people all over the world, with an accompanying economic loss of up to 100 trillion dollars.^[^ ^1^ ^]^ There are two traditional approaches to deal with the infection threats.^[^ ^2^, ^3^ ^]^ The first approach is constructing antifouling surfaces via hydrophilic polymers or superhydrophobic structure to fend off bacteria adhesion, which presents good biocompatibility.^[^ ^4^, ^5^ ^]^ The second approach is utilizing bactericides to actively kill the bacteria, which owns high bactericidal efficiency.^[^ ^6^ ^]^ However, the “repelling” approach is incapable to inhibit bacterial proliferation. The “killing” approach may cause cytotoxicity and drug‐resistant bacteria which limit its application.^[^ ^7^ ^]^ Furthermore, the aggregation of dead microorganisms on the surface block antibiotic functional groups, and may lead to the immune and inflammatory response.^[^ ^8^, ^9^ ^]^

Stimuli‐responsive antibacterial materials are sensitive to signals of the external environment or the pathological abnormalities, which can regulate the conflicts between biocompatibility and high bactericidal efficiency by switching on demand,^[^ ^10^, ^11^ ^]^ can detect infections timely, reduce or avoid the generation of drug‐resistant bacteria via controlled release or exposure antibiotics,^[^ ^12^ ^]^ and inhibit the formation of bacterial biofilms at the early stage of infections.^[^ ^13^, ^14^ ^]^ Therefore, stimuli‐responsive materials are suitable for antibacterial applications, that have been widely used in medical devices, drug delivery, diagnostics, and tissue engineering.^[^ ^15^, ^16^ ^]^ However, to the best of our knowledge, few studies have summarized and categorized stimuli‐responsive antibacterial materials systematically and comprehensively. Although some stimuli‐responsive materials have been reviewed, they mainly focus on a single stimulus or single application.^[^ ^17^, ^18^, ^19^, ^20^ ^]^ Few reviews research the stimuli‐responsive materials for antibacterial study depending on the various sources of stimuli systematically.^[^ ^21^, ^22^ ^]^ Bacteria are traditionally classified into Gram‐positive and Gram‐negative bacteria based on the difference of cell walls.^[^ ^23^ ^]^ Gram‐positive bacteria are bounded by a single‐unit lipid membrane and a thick layer of peptidoglycan, such as *Staphylococcus aureus* (*S. aureus*). Gram‐negative bacteria are composed of a thin peptidoglycan cell wall sandwiched between an inner cytoplasmic cell membrane and a bacterial outer membrane, like *Escherichia coli* (*E. coli*). The design principles of stimuli‐responsive materials against different bacteria are varied, few studies have focused on this issue.

In this review, we first classify the antibacterial stimuli into physical stimuli and bacterial metabolites stimuli. Physical stimuli regulate the bactericidal property in response to the external environmental signals, such as temperature, light, electricity, salt, and so on. Bacterial metabolites stimuli favor self‐adaptive antibacterial activity, include acid, enzyme, redox.^[^ ^24^ ^]^ Then, we summarize the fundamental structural features, the response mechanisms, design principles of stimuli‐responsive antibacterial materials. The structure of stimuli‐responsive materials can be constructed with different sensitive groups. For instance, PEG analogs achieve different transition temperature by adjusting the group composition; salt‐responsive materials commonly contain anionic and cationic ionizable units; protonated amino groups and Schiff base bond have been widely used as acid‐sensitive groups; enzyme‐responsive materials cannot be connected without enzyme‐sensitive specific chemical bonds. The response mechanisms and design rationales are closely related to the sources of stimuli and the categories of bacteria, which are explained by the detailed examples in the following discussions. Finally, a range of biomedical applications is discussed, including medical devices, drug delivery, theranostics, tissue engineering, and so on. Furthermore, why the stimuli‐responsive antibacterial materials can inhibit the formation of biofilm and the emergence of resistant bacteria are discussed extensively in each part. This review will provide scientific guidance to facilitate the clinical applications of stimuli‐responsive antibacterial materials and promote the development of personalized medicine.

## 2 Stimuli‐Responsive Antibacterial Materials

**Figure 1:**
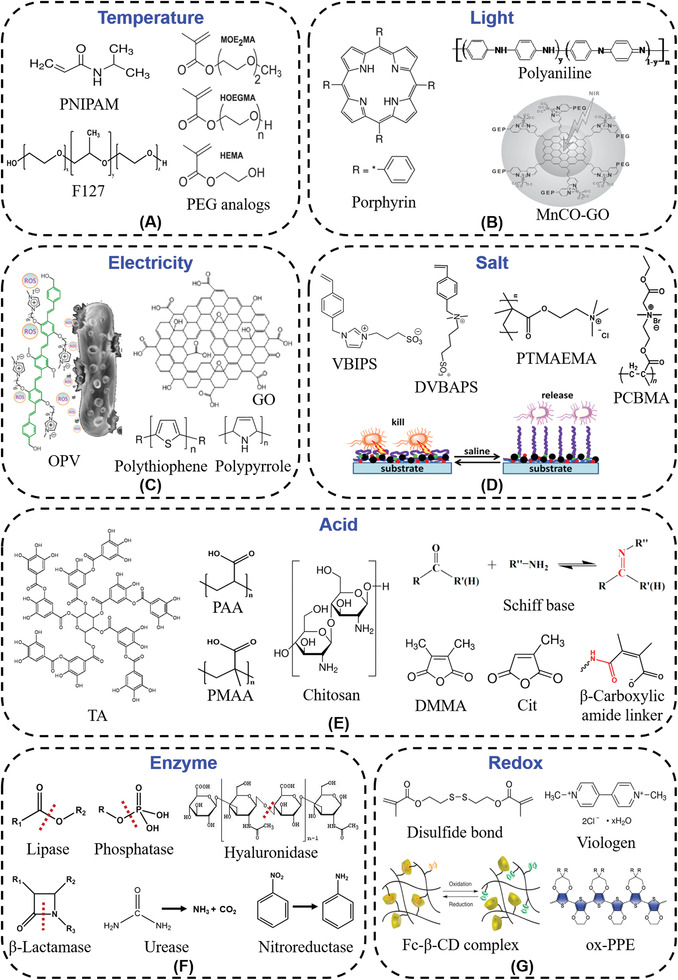
Molecular structures of typical stimuli‐responsive materials. A) Thermo‐responsive materials. Reproduced with permission.^[^ ^34^ ^]^ Copyright 2010, Wiley‐VCH. B) Light‐responsive materials. Reproduced with permission.^[^ ^47^ ^]^ Copyright 2015, Wiley‐VCH. C) Electro‐responsive materials. Reproduced with permission.^[^ ^49^ ^]^ Copyright 2018, American Chemical Society. D) Salt‐responsive materials. Reproduced with permission.^[^ ^59^ ^]^ Copyright 2020, Elsevier. E) Acid‐responsive materials. Reproduced with permission.^[^ ^69^ ^]^ Copyright 2015, American Chemical Society. F) Enzyme‐responsive materials. G) Redox‐responsive materials. Reproduced with permission.^[^ ^87^ ^]^ Copyright 2011, Nature Publishing Group.

According to the sources of the stimuli, stimuli‐responsive materials can be divided into two categories: materials that respond to physical stimuli (e.g., temperature, light, electricity, salt, etc.) and materials that respond to bacterial metabolites stimuli (e.g., acid, enzyme, redox, etc.). The physical responsive materials are mostly designed by adjusting the entire molecular structure. The bacterial metabolites responsive materials are a response to the stimuli via specific chemical bonds, like acid and enzyme sensitive bonds. Recently, multiple stimuli response materials have been developed via combination of polymers with different functional groups. The basic structural features of different stimuli‐responsive materials are displayed in **Figure** **1**.

### 2.1 Physical Stimuli‐Responsive Antibacterial Materials

Physical responsive materials are sensitive to external environmental signals, like temperature, light, electricity, salt, etc. (Figure 1).^[^ ^25^ ^]^ The materials that have transition temperature between room temperature and body temperature are widely used as thermo‐responsive materials in biological field.^[^ ^26^ ^]^ The light‐responsive materials include three categories, photodynamic therapy (PDT), photothermal therapy (PTT), and photo‐induced gas therapy.^[^ ^27^, ^28^, ^29^ ^]^ Electro‐responsive antibacterial materials must consist of conductive structure. Salt‐responsive materials commonly contain anionic and cationic ionizable units.^[^ ^30^ ^]^ When designing physical responsive materials, easy‐to‐control stimuli are often considered. For example, thermo‐responsive materials have been widely used in biomedical field, which takes advantage of the temperature difference between body's internal and external environment. Light and electricity are convenient to operate with high stability.

Thermo‐responsive materials can be divided into two categories, water‐insoluble above the lower critical solution temperature (LCST) and water‐insoluble below the upper critical solution temperature.^[^ ^31^ ^]^ Materials with transition temperature close to body temperature is suitable for biomedical applications. Poly(*N*‐isopropylacrylamide) (PNIPAM) is the most commonly used thermo‐responsive polymer, which can transform reversibly from a hydrophilic coil state to a hydrophobic globule state near 32 ℃ by adjusting the water solubility (Figure 1A).^[^ ^32^, ^33^ ^]^ The PEG analogs based on 2‐(2‐methoxyethoxy)ethyl methacrylate (MEO_2_MA), oligo(ethylene glycol) methacrylate (OEGMA), and 2‐hydroxyethyl methacrylate (HEMA) copolymers are other thermo‐responsive polymers (Figure 1A).^[^ ^34^ ^]^ Compared with PNIPAM, these copolymers present an LCST less sensitive to surrounding conditions. By adjusting the content and length of OEGMA chains, the average collapse temperature of these brushes can be successfully turned from 22 to 40 ℃.^[^ ^35^ ^]^ Besides, Pluronic F127 (PF127, also known as Poloxamer 407) is a thermo‐responsive gel that presents gelling property and proper sol‐gel transition temperature (Figure 1A). Along with the temperature rise, PF127 micelles form a gel. It can be injected in the liquid state and gelled in situ at body temperature, which has applications in drug‐depot and delivery systems.^[^ ^36^, ^37^ ^]^ The challenges of thermo‐responsive materials are the complicated process to adjust the transition temperature of PEG analogs and the limited functionality of PNIPAM. To achieve the multi‐functionality of thermo‐responsive materials and satisfy the biomedical applications in various situations, some research has co‐polymerized functional monomers with thermo‐responsive materials.

Light‐responsive materials usually consist of chromophores that can transform light inputs into potential chemical or energy outputs. Light‐responsive antibacterial research can be divided into antimicrobial PDT, antimicrobial PTT,^[^ ^38^ ^]^ and antimicrobial photo‐induced gas therapy. Light is non‐invasive, which can be imposed instantly with high delivery precision compared to other stimuli.^[^ ^39^, ^40^ ^]^ The advantages of light‐responsive antibacterial materials are not inducing drug‐resistance and killing drug‐resistant bacteria, relying on the bactericidal mechanisms. Porphyrins and their derivatives are commonly used PDT photosensitizers (PSs) which have intense visible‐light absorption and high singlet oxygen quantum yield (Figure 1B). Chlorine e6 (Ce6) is a porphyrin analog PS, always uses in the visible light‐triggered delivery system and shows therapeutic effects by producing reactive oxygen species (ROS) such as singlet oxygen (^1^O_2_).^[^ ^41^ ^]^ But some PSs have cytotoxicity. Chlorophyll is a popular PS that has excellent biocompatibility and high singlet‐oxygen quantum yields.^[^ ^42^ ^]^ About PTT, gold nanoparticles (AuNPs), graphene, carbon nanotubes, magnetic nanoparticles, and other inorganic materials can absorb photons in the near‐infrared radiation (NIR) region and transform light energy into thermal energy. AuNPs can be used to control drug delivery and stimulate tissue reconstruction, which are inert, nontoxic, and have adjustable optical and photothermal conversion properties.^[^ ^43^ ^]^ Polyaniline (PANI) has high photothermal conversion efficiency depending on the conjugated benzenoid or quinonoid units (Figure 1B).^[^ ^44^ ^]^ Recently, more and more researchers pay attention to photo‐induced gas therapy. Gas can directly change disease status and influence diverse physiological and pathophysiological processes, such as nitric oxide (NO), carbon monoxide (CO), and hydrogen sulfide (H_2_S).^[^ ^45^, ^46^ ^]^ The challenges of gas therapies are efficient gas delivery and controllable gas release.^[^ ^29^ ^]^ Gas releasing molecules or polymers have emerged to realize controlled release. Light‐responsive nanomedicine (MnCO‐graphene oxide (GO)) that cages Mn‐carbonyl carbon monoxide releasing molecules in a small GO nanosheet, achieves the on‐demand release of CO and evades the risk of CO poisoning (Figure 1B).^[^ ^47^ ^]^

Electro‐responsive materials are increasingly important that can be applied in drug delivery, sensors, smart actuators toward soft robotics, and artificial muscles.^[^ ^48^ ^]^ Cationic oligo(p‐phenylenevinylene) (OPV) can be used in the electric‐driven luminous system based on electrochemiluminescence (ECL) to kill pathogenic bacteria (Figure 1C). When energy transfers from ECL to OPV, the negative membrane of pathogenic bacteria will be adsorbed to the cationic OPV via electrostatic and/or hydrophobic interactions and the generated ROS will kill them simultaneously.^[^ ^49^ ^]^ Graphene is a commonly used conductive material. Graphene oxide‐graft‐quaternized CS nanohybrid electrode coating can achieve at least 99.9999% killing (i.e., 6 log reduction) of *E. coli* in biocontaminated water by capacitive deionization disinfection process.^[^ ^50^, ^51^ ^]^ Moreover, many other conductive polymers have been widely applied in biomedical applications, such as PANI, polythiophene, and polypyrrole and their various derivatives (Figure 1C).^[^ ^52^ ^]^

Salt‐responsive materials mainly adopt bacterial killing‐release strategy to prevent the attachment of bacteria and formation of biofilm.^[^ ^53^ ^]^ Compared with contact‐killing and anti‐adhesion/bacteria repelling strategies, this strategy realizes the reversible switch between killing bacteria and releasing dead bacteria.^[^ ^54^ ^]^ Zwitterionic polymers possess anionic and cationic ionizable units, are notable neutral, hydrophilic, salt‐sensitive polymers.^[^ ^55^ ^]^ There are many kinds of zwitterionic polymer brushes with different structures, such as poly(3‐(1‐(4‐vinylbenzyl)‐1H‐imidazol‐3‐ium‐3‐yl) propane‐1‐sulfonate) (polyVBIPS),^[^ ^56^, ^57^ ^]^ poly(3‐(dimethyl(4‐vinylbenzyl) ammonio) propyl sulfonate) (polyDVBAPS),^[^ ^58^ ^]^ poly(3‐(dimethyl(4‐vinylbenzyl)ammonio)butanesulfonate) (polyDVBABS)^[^ ^59^ ^]^ and so on (Figure 1D). In addition, cationic poly((trimethylamino)ethyl methacrylate chloride) (PTMAEMA) modified surface can contact kill the attached bacteria efficiently. And the bacterial release and regeneration of antimicrobial surface are obtained by washing with electrolyte solutions containing anions simply (Figure 1D). The carboxybetaine esters achieve the switch between bactericidal performance to antifouling property and releasing the dead bacteria, when the cationic derivatives are hydrolyzed to nonfouling zwitterionic polymers upon washing with salt solution, like poly(*N*,*N*‐dimethyl*N*‐(ethoxycarbonylmethyl)‐*N*‐[2′‐(methacryloyloxy)ethyl]‐ammonium bromide) (PCBMA) (Figure 1D).^[^ ^60^, ^61^ ^]^ The major challenge of salt‐responsive materials is that they are vulnerable to environment, like pH of the solution, concentration of electrolytes. While the obvious advantage of salt‐responsive materials is convenient to realize switch between bacterial killing and bacterial releasing.

### 2.2 Bacterial Metabolites Stimuli‐Responsive Antibacterial Materials

Materials that respond to bacterial metabolites stimuli are favoring self‐adaptive antibacterial systems and precision medicine. Bactericides can be precisely released or exposed on demand, that is, only when and where needed, which can avoid the overuse of bactericides and reduce the generation of drug‐resistant bacteria.^[^ ^62^ ^]^ Acid‐responsive materials include acrylic polymers, Schiff base bond, and *β*‐carboxylic amide bond. Enzyme‐responsive materials depend on the types of enzyme secreted by different bacteria, and own the characteristic of specificity.^[^ ^63^ ^]^ Redox‐responsive materials are the materials capable of redox reaction.

Acid‐responsive materials can respond to bacterial acids like acetic acid, lactic acid, malic acid. In acid‐responsive systems, poly(acrylic acid) (PAA), poly(methacrylic acid) (PMAA), tannic acid (TA), and chitosan (CS) are widely used due to good biocompatible (Figure 1E).^[^ ^64^, ^65^, ^66^ ^]^ They can bind protonated amino groups of antibiotics or cationic fungicides electrostatically, and release the fungicides in response to the acidic microenvironment.^[^ ^67^ ^]^ Not only the hydrophilic biocides, but the hydrophobic antibiotic triclosan can also be loaded into the CS and PAA coating by cationic micelles.^[^ ^68^ ^]^ But the stability of electrostatic interactions is poor. To improve the stability, acid response chemical bonds are developed. Schiff base bond is a dynamic covalent bond formed by the reaction between aldehyde (or ketone) and amino groups, that can hydrolyze under a weakly acidic condition (Figure 1E).^[^ ^69^, ^70^ ^]^ Gentamicin sulfate is grafted onto aldylated sodium alginate via Schiff base reaction, released in response to the bacterial acidification, and killed more than 99.99% of bacteria in 24 h.^[^ ^71^ ^]^ The dimethylmaleic anhydride (DMMA) and citraconic amide (Cit), which are *α*‐methyl derivatives of maleic anhydride, can provide a pH‐dependent degradability to release drugs or achieve charge conversion (Figure 1E). The reaction between primary amines and DMMA or Cit forms a *β*‐carboxylic amide linker, and keeps stable at neutral pH, while hydrolyzes at acid pH.^[^ ^72^ ^]^ The characteristics of *β*‐carboxylic amide exactly match the bacterial acidic metabolism, and have a potential application in antibacterial field.^[^ ^73^, ^74^ ^]^

Enzyme‐responsive materials with high selectivity and catalytic efficiency have been widely employed for the design of antibacterial materials.^[^ ^17^, ^75^ ^]^ Many enzymes such as lipase, phosphatase, hyaluronidase, *β*‐lactamase, serine protease, urease, gelatinase, and nitroreductase have been used in stimuli‐responsive antibacterial theranostics (Figure 1F).^[^ ^76^, ^77^, ^78^ ^]^ The enzymes catalyze reactions via the reduction/oxidation of substrates and the formation/cleavage of chemical bonds. Enzyme‐responsive materials achieve the detection of bacterial infection, the controlled release of antibiotics, better biocompatibility of antimicrobial peptides, and killing the drug‐resistant bacteria selectively.^[^ ^79^, ^80^, ^81^ ^]^ Owing to *β*‐lactam antibiotics have been widely used in hospital, a series of *β*‑lactamase activated antibacterial prodrugs are prepared to overcome the bacterial resistance to *β*‐lactam antibiotics.^[^ ^82^, ^83^ ^]^ Some *β*‑lactamase activated probes are also developed to label and distinguish the *β*‐lactam resistant bacteria.^[^ ^84^ ^]^ Enzyme‐responsive materials have great promise to realize specific and selective antibacterial theranostics.

Redox‐responsive materials can respond to oxidants or reductants generated by bacteria, which have been used in various biomedical applications. In the process of redox response, major chemical groups include disulfide bonds, organometallic complexes, viologens, and tetrathiafulvalene (Figure 1G).^[^ ^85^ ^]^ Glutathione and cysteine is the most abundant reducing agent in many cells. It has an apparent difference between intracellular and extracellular concentration, that can be used to design redox‐responsive drug delivery systems.^[^ ^86^ ^]^ The redox state of ferrocene (Fc) increased the binding affinity to beta‐cyclodextrin (*β*‐CD). The host‐guest interactions between the pendant complimentary Fc and *β*‐CD allowed controlled self‐assembly into a hydrogel (Figure 1G).^[^ ^25^, ^87^ ^]^ In addition, the polymer ox‐PPE (poly(3,4‐propylenedioxythiophen‐alt‐3,4‐ethylenedioxythiophene) copolymer) with electrochromic property is developed for rapid and convenient bacterial detection and identification (Figure 1G).^[^ ^88^ ^]^

## 3 Design Principles

Design principles of stimuli‐responsive antibacterial materials depend on the types of stimuli. Different responsive functional groups can be integrated into the responsive materials to achieve different responsive antibacterial effects. The detailed design principles are explained according to the stimuli in the following sections.

### 3.1 Design Principles of Physical Stimuli‐Responsive Antibacterial Materials

The design principles of physical stimuli‐responsive antibacterial materials depend on different sources of stimuli. The most important design principle of thermo‐responsive materials is maintaining the transition temperature between room temperature and body temperature. So that the materials can obtain the transition easily along with good biocompatibility. The most important constituent of light‐responsive materials is the photosensitive group, which decides the absorption wavelength. How to use the conductivity of the material to detect or kill bacteria is critical for electro‐responsive materials. The obvious characteristic of salt‐responsive materials is the coordination of positive and negative groups.

#### 3.1.1 Temperature

**Figure 2:**
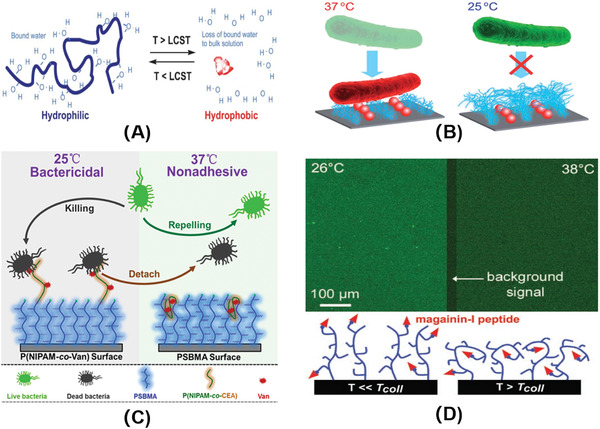
A) Schematic of smart polymer response to temperature. Reproduced with permission.^[^ ^91^ ^]^ Copyright 2005, Royal Society of Chemistry. B) Schematic illustration of interactions between bacteria (*E. coli*) and nanopatterned PNIPAAm/Lys surface at different temperatures. Reproduced with permission.^[^ ^93^ ^]^ Copyright 2014, Royal Society of Chemistry. C) Schematic diagram of the thermo‐responsive hierarchical antibacterial surface. Reproduced with permission.^[^ ^96^ ^]^ Copyright 2017, American Chemical Society. D) Fluorescence images of (biotinyl‐MAG‐Cys)‐grafted P(MEO_2_MA_50_‐HOEGMA_20_‐HEMA_30_) brushes incubated at 26 and 38 °C in streptavidin FITC solution (top), and schematic drawing of the brush conformation well below and slightly above *T*_coll_ (bottom). Reproduced with permission.^[^ ^34^ ^]^ Copyright 2010, Wiley‐VCH.

Implantable or interventional medical devices need bactericidal activities when exposed in the hospital environment, such as operation process or the part used in the external environment.^[^ ^89^ ^]^ And biocompatibility is necessary during use under physiological conditions. Considering the temperature difference between body's internal and external environment, thermo‐responsive materials have been extensively studied. PNIPAM is the most common thermo‐responsive polymer with LCST between body temperature and room temperature.^[^ ^90^ ^]^ The origin of the phase transition is the entropic gain along with the increasing temperature. When the temperature is lower than the LCST of PNIPAM, the intermolecular hydrogen bonds play a major role, and the molecular chains stretch and become hydrophilic; when the temperature is higher than its LCST, the intramolecular hydrogen bonds play a major role, and the molecular chains collapse and become hydrophobic (**Figure** **2**A).^[^ ^91^, ^92^ ^]^ PNIPAM was used to fabricate thermo‐responsive surfaces which could regulate cell adhesion and antibacterial activity. PNIPAM and bactericides (quaternary ammonium salts or lysozyme) were grafted on the surface (Figure 2B).^[^ ^93^, ^94^ ^]^ At body temperature, PNIPAM brushes collapsed and exposed bactericide to kill the adherent bacteria, and the bacterial killing ratio was about 90%; at room temperature, PNIPAM brushes stretched to achieve antifouling function and released about 85% adherent bacteria.^[^ ^95^ ^]^ But current PNIPAM‐based antibacterial surfaces most own bactericidal activity at body temperature during use and exhibit bacterial repellency at room temperature, which is not meet the actual applications.

In order to meet the needs of practical applications, a thermo‐responsive bilayer surface that transformed from bactericidal to antifouling along with the rise of temperature was constructed (Figure 2C).^[^ ^96^ ^]^ When stored at room temperature (lower than the LCST of PNIPAM), PNIPAM molecular chain stretched to expose vancomycin to kill the adherent bacteria on the surface. The killing efficiency of this bilayer surface reached 88.6%. When used under body temperature which was higher than the LCST of PNIPAM, the PNIPAM molecular chain collapsed and wrapped the antibiotic to block the bactericidal function. The wrapping of antibiotic greatly reduced the generation of resistant bacteria. Meanwhile, the hydrophilic PSBMA brush extended, and covered the upper layer of P(NIPAM‐co‐Van) hydrophobic copolymer brushes, that could repel ≈80% adherent bacteria and show good biocompatibility. The antifouling surface inhibited the adhesion of planktonic bacteria, thereby interfering with the earliest stages of biofilm formation. And the surface could release about 72.5% of previously attached dead bacteria with the change of temperature, showing “self‐cleaning” performance. Most importantly, this study realizes the bactericidal activities at room temperature and presents bacterial repellency at physiological temperature via a hierarchical polymer architecture. Simultaneously it provides new ideas to increase the multi‐functionality of PNIPAM.

Polymers containing H‐bonding sites for water molecules and the *N*,*N*‐diethylacrylamide copolymer can also exhibit an LCST phenomenon between 25 and 37 ℃. Glinel's group^[^ ^34^ ^]^ developed thermo‐sensitive switchable surfaces by adjusting the composition of PEG analogs (Figure 1A). When at use temperature (37 ℃), the copolymer molecular brush collapsed and wrapped the antibacterial peptides, and the short hydrophilic chains on the surface achieved excellent antifouling effect. At storage temperature (25 ℃), the polymer brush stretched, exposed antibacterial peptide, which could kill the adherent bacteria (Figure 2D). The immobilized antimicrobial peptides were tagged with fluorescein, and were wrapped ≈40% at 38 °C and ≈60% at 42 °C when in comparison with the fluorescence intensity at 26 °C. This study synthesizes a kind of copolymer to realize bactericidal effect at lower temperature, and resists bacterial adhesion at higher temperature.

#### 3.1.2 Light

As a convenient non‐invasive on/off trigger, light has advantages of accurate control of irradiation site, time, dosage, facile operation, and not being affected by the surrounding environment such as pH, ionic strength, temperature, etc.^[^ ^97^ ^]^ Light‐responsive materials have been widely used in antibacterial field. At present, the main light‐controlled antibacterial strategies include: antimicrobial PDT, antimicrobial PTT, and antimicrobial photo‐induced gas therapy.^[^ ^98^, ^99^ ^]^ More importantly, PDT, PTT, and antimicrobial photo‐induced gas therapy will not induce drug‐resistance and can kill the drug‐resistant bacteria.

**Figure 3:**
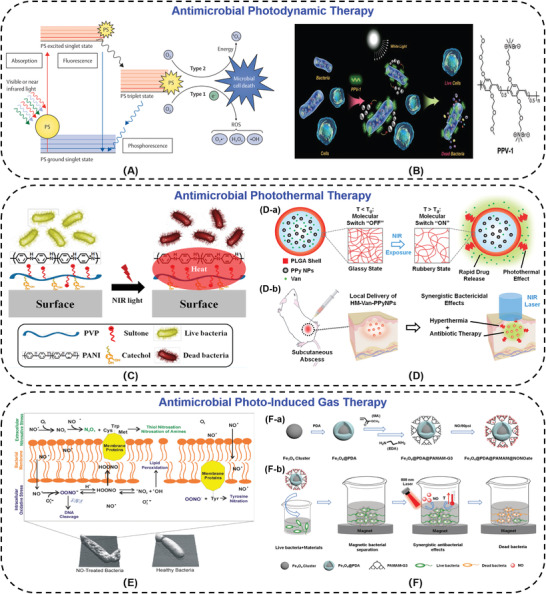
A) Mechanism of photo‐antimicrobial action. Reproduced with permission.^[^ ^101^ ^]^ Copyright 2017, Elsevier. B) Schematic representation of PPV‐1 for the selective recognition, imaging, and killing of bacteria over mammalian cells and the chemical structure of PPV‐1. Reproduced with permission.^[^ ^112^ ^]^ Copyright 2011, Wiley‐VCH. C) Illustration of the PANI:PVP coating photo‐thermolysis bacteria via NIR irradiation. Reproduced with permission.^[^ ^116^ ^]^ Copyright 2015, American Chemical Society. D) Schematic illustrations of the dual‐modality photothermal/antibiotic treatments for infected tissues of hollow microspheres containing vancomycin and polypyrrole nanoparticles. D‐a) Photothermally‐responsive drug release. D‐b) Dual‐modality of photothermal/antibiotic therapies for treating infected tissues. Reproduced with permission.^[^ ^117^ ^]^ Copyright 2015, Elsevier. E) The antibacterial mechanisms of nitric oxide and its byproducts. Reproduced with permission.^[^ ^118^ ^]^ Copyright 2012, Royal Society of Chemistry. F) A nanocomposite synergistic photo‐thermolysis and NO antibacterial properties. F‐a) Synthetic route of Fe_3_O_4_@PDA@PAMAM@NONOate. F‐b) Fe_3_O_4_@PDA@PAMAM@NONOate for magnetic separation, synergistic photothermal, and NO killing of bacteria. Reproduced with permission.^[^ ^124^ ^]^ Copyright 2018, Wiley‐VCH.

Antimicrobial PDT is employed with a light source, oxygen, and PS, that could control the treatment spatiotemporally.^[^ ^100^ ^]^ Upon light irradiation, PS rapidly forms their triplet excited states and then transfers to ground state (triplet) molecular oxygen to generate ROS, such as free radical and singlet oxygen (**Figure** **3**A).^[^ ^27^, ^101^, ^102^ ^]^ ROS kill bacteria by interacting with cellular components and causing critical damage to DNA, proteins, and membranes.^[^ ^103^, ^104^, ^105^ ^]^ Thus ROS has broad‐spectrum antimicrobial activity and efficient inactivation of drug‐resistant bacteria.^[^ ^106^, ^107^ ^]^ Porphyrin family is a commonly used PS and can be used to treat tumors, cardiovascular diseases, skin diseases, and eye diseases, which have great potential in biomedical applications.^[^ ^42^, ^108^, ^109^ ^]^ However, due to the complex outer membrane structure of Gram‐negative bacteria which without membrane disrupting agent (such as CaCl_2_, EDTA, or polymyxin B nonapeptide), the bactericidal property of porphyrin to Gram‐negative bacteria is weak. Carvalho et al.^[^ ^110^ ^]^ synthesized cationic porphyrins that could attract bacteria through electrostatic interactions, and had broad‐spectrum bactericidal properties. 20 µM prepared cationic porphyrins could inactivate almost 100% infected *E. coli* upon 90 min irradiation with white light of 43.2 J cm^−2^. Xu's group^[^ ^111^ ^]^ synthesized an eosin Y‐based antibacterial polycation polymer which contained quaternary ammonium, PS, primary amine, and hydroxyl species. The polymer could be readily coated on different substrates, and had good antifouling capability, high photodynamic killing efficiency under light irradiation, and good biocompatibility. The minimum inhibitory concentration (MIC) against *S. aureus* was about 16 µg mL^−1^ upon 30 min irradiation with wavelength of 520 ± 10 nm and a power density of 60 mW cm^−2^. Wang's group^[^ ^112^ ^]^ recently synthesized a multifunctional cationic poly(p‐phenylene vinylene) polyelectrolyte (PPV‐1) that could selectively identify and kill bacteria in mammalian cells (Figure 3B). PPV‐1 (10 µM) killed more than 90% of infected bacteria under 6 min illumination with 75 mW cm^−2^ white light. The bacterial cell walls are composed principally of peptidoglycans, which are negative charges. For mammalian cells, the negatively charged phosphatidylserine is mainly located in the inner side of plasma membrane, reducing the negative charge density to approximate electrical neutrality. Therefore, cationic PPV‐1 could selectively bind to the surface of bacteria through electrostatic interaction, thus killing bacteria over mammalian cells. Besides, the PEG side chains of PPV‐1 further prevented non‐specific adsorption to mammalian cells. The characteristics of PDT that can kill drug‐resistant bacteria and not induce bacterial resistance are attractive to fight against resistant infections. The trait of targeting bacteria among mammalian cells can reduce the cytotoxicity of ROS.

In recent years, there has been an increasing interest in developing PTT as a powerful and alternative tool to fight against pathogenic bacteria by denaturation of their proteins, including drug‐resistant bacteria.^[^ ^113^ ^]^ NIR (700–1100 nm) can penetrate the skin for 10 mm deep without causing damage to blood vessels and normal tissues, which is a suitable wavelength region for PTT.^[^ ^114^, ^115^ ^]^ PANI, polypyrrole (PPy), poly(3,4‐ethylenedioxythiophene)‐poly(styrene sulfonic acid), graphene, gold nanoparticles, carbon nanotubes, magnetic nanoparticles, and other inorganic materials, can absorb the energy of NIR light and convert into thermal energy to kill bacteria. The proteins are denatured along with the increasing temperature (≥50 ℃). The PANI modified surface could kill 99.9% of the Gram‐positive and ‐negative bacteria within 3 min under NIR illumination (Figure 3C).^[^ ^116^ ^]^ Sung's group constructed the hollow microspheres (HM‐Van‐PPyNPs) with poly(D,L‐lactic‐co‐glycolic acid) (PLGA) shell and vancomycin (Van) and polypyrrole nanoparticles (PPy NPs) core. PPy NPs could absorb the NIR laser and convert the light energy into thermal energy. This photothermal conversion not only killed bacteria at the site of abscesses, but also increased the mobility of shell chains to release vancomycin to enhance the therapeutic efficacy synergistically (Figure 3D).^[^ ^117^ ^]^ The bactericidal efficiency of samples comprised of Van and PPy NPs (100 mg mL^−1^ in saline which contained 10 mg Van mL^−1^, 100 µL) was ≈80% against methicillin‐resistant *S. aureus* upon irradiation by the NIR laser (0.5 W cm^−2^ for 15 min).

Some gas signal molecules such as NO, have nitrosative and oxidative action upon reaction with oxygen or reactive oxygen intermediates. NO has been demonstrated to interact with bacterial proteins, DNA, metabolic enzymes, and cell membranes to influence diverse physiological and pathophysiological processes (Figure 3E).^[^ ^118^, ^119^ ^]^ CO can kill bacteria, including drug‐resistant bacteria, by inhibiting the bacterial respiratory chain, enhancing the generation of ROS, and interfering gene expression.^[^ ^120^, ^121^, ^122^ ^]^ However, the cytotoxicity of gas at high concentrations limits their applications.^[^ ^28^, ^123^ ^]^ The key to using gas for bactericidal therapy is to find a suitable carrier and achieve controllable release. Xue's group^[^ ^124^ ^]^ exploited the NO‐releasing dendritic Fe_3_O_4_@PDA@PAMAM@NONOate nanocomposites to deliver NO. Under NIR irradiation, the nanospheres could convert light to thermal energy and stimulate the release of NO (Figure 3F). Based on the synergistic effect of NO and heat, the nanocomposites (0.25 mg mL^−1^) could effectively kill almost all drug‐resistant bacteria with 808 laser irradiation (0.5 W cm^−2^, 5 min), avoid the generation of bacterial drug‐resistance, and prevent the formation of bacterial biofilms. Based on accomplishing effective gas delivery and gas controlled release gradually, gas therapies have been developed rapidly in recent years.

Light source is convenient to operate, light‐responsive antibacterial materials have the ability to kill drug‐resistant bacteria and are unlikely to induce bacterial resistance. While the ROS, high temperature, and therapeutic gas have a little cytotoxicity. Targeting function can kill bacteria among mammalian cells selectively and improve bactericidal effect. On the other hand, diagnostic function can indicate the dosage, duration, and location of bacterial infections, rendering light‐responsive sterilization more controllable. Thus, integrating targeting and diagnostic function with light‐controlled antibacterial strategies are new trends of development.

#### 3.1.3 Electricity

**Figure 4:**
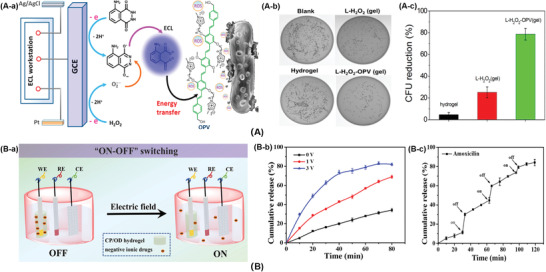
A) The ECL‐therapeutics. A‐a) Electric‐driven mechanism to produce ROS and kill pathogenic bacteria. A‐b) Plate photographs for C. *albicans* treated with different hydrogel systems. A‐c) Antibacterial activity of the hydrogels. Reproduced with permission.^[^ ^49^ ^]^ Copyright 2018, American Chemical Society. B) The conductive hydrogel to control the release of bactericidal drugs. B‐a) Schematic illustration of pulse release of drug model from conductive hydrogel. B‐b) Amoxicillin release in PBS under different electric potentials. B‐c) Amoxicillin release in PBS under an electric potential of 3 V for 3 min, repeated every 60 min. Reproduced with permission.^[^ ^127^ ^]^ Copyright 2018, Elsevier.

Electrical stimulation has characteristics of high‐level spatial and time controllability, rapid and reverse induction, and non‐invasiveness.^[^ ^125^, ^126^ ^]^ Based on electrochemical luminescence (ECL) electro‐driven luminescence system, Wang's group^[^ ^49^ ^]^ synthesized a PS that could be excited to produce ROS to realize the antibacterial function (**Figure** **4**A). The specific research mechanism was relying on the perfect spectral overlap and energy transfer of luminol and PS, which could convert the surrounding oxygen molecules into ROS. In particular, 5‐amino‐2,3‐dihydro‐1,4‐naphthalenedione was used as ECL reagent, in the presence of H_2_O_2_ in alkaline solution, its emission wavelength was 400–550 nm. The cationic PS oligomer (p‐phenylene vinylene) (OPV) had a relatively broad absorption (350–550 nm) and a maximum emission wavelength of 550 nm. OPV absorbed the energy of ECL to excite the surrounding oxygen molecules to produce more ROS for sterilization. The bactericidal efficiency of the luminol‐H_2_O_2_‐OPV (L‐H_2_O_2_‐OPV) hydrogel toward *C. albicans* was about 80%, while the control group L‐H_2_O_2_ hydrogel exhibited low efficiency (25%), and the hydrogel itself was about 5% (Figure 4A(b,c)). This strategy overcomes the problem of the limited penetration depth of light, and could realize the possibility of sterilization in deeper tissue treatment. Furthermore, electric field was able to control the release of bactericidal drugs in conductive hydrogel (Figure 4B). Negatively charged amoxicillin, ibuprofen, aminoglycosides, etc. were enveloped in conductive hydrogel prepared by CS‐graft‐PANI (CP) copolymer and oxidized dextran (OD). Electricity could reduce PANI, and the CP/OD hydrogel with positive charge was reduced, thus the charged drugs were expelled from the hydrogel. At the same time, electric field could drive the charged drugs to migrate to the electrode, which results in the release of the drugs.^[^ ^127^ ^]^ As shown in Figure 4B(b), with the increase of the voltage, the cumulative release of amoxicillin significantly increased. The conductive hydrogel could exhibit a kind of “on‐off” pulse release upon applying a voltage (Figure 4B(c)). In electric response antibacterial systems, conductive polymers, conductive ions, or other conductive materials are essential.

#### 3.1.4 Salt

**Figure 5:**
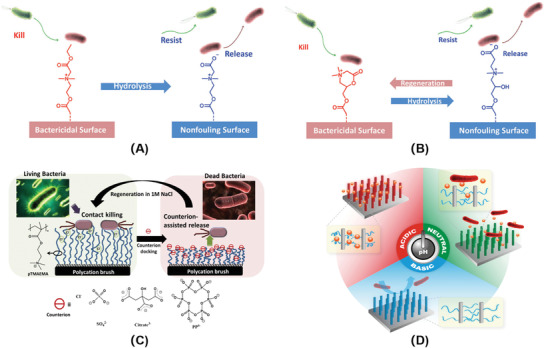
A) A bactericidal surface based on a zwitterionic ester precursor which killed bacteria at the cationic state and then underwent hydrolysis to release dead bacterial cells. Reproduced with permission.^[^ ^129^ ^]^ Copyright 2014, Wiley‐VCH. B) A switchable material undergoing reversible lactonization that prevented bacterial surface proliferation at its cationic state and released inactivated bacterial cells at its zwitterionic state. Reproduced with permission.^[^ ^129^ ^]^ Copyright 2014, Wiley‐VCH. C) Schematic illustration of contact killing and counterion‐assisted release of bacteria on poly((trimethylamino)ethyl methacrylate chloride) surface. Reproduced with permission.^[^ ^132^ ^]^ Copyright 2015, American Chemical Society. D) Schematic illustration of a smart antibacterial surface with pH‐responsive capability of loading biocide, killing bacteria, and releasing bacteria. Reproduced with permission.^[^ ^133^ ^]^ Copyright 2016, Wiley‐VCH.

The salt‐responsive adjustment is a relatively mild method and would not cause obvious adverse effects on the environment.^[^ ^56^, ^128^ ^]^ A class of switchable surfaces, which could switch between bacterial killing and bacterial releasing triggered by salt was developed.^[^ ^129^ ^]^ Cationic bactericides, like quaternary ammonium compounds and antimicrobial peptides, could efficiently kill a variety of microorganisms, while not affording the requirements of biocompatibility.^[^ ^130^ ^]^ Jiang's group^[^ ^131^ ^]^ first synthesized the cationic ester that was able to kill bacterial cells effectively, then switched to a zwitterionic biocompatible surface and released dead bacterial cells upon hydrolysis of ester groups by adding salt (**Figure** **5**A). Owning to the hydrolysis of ester groups was not reversible, subsequently, Jiang^[^ ^7^ ^]^ designed a cationic *N*,*N*‐dimethyl‐2‐morpholinone (CB‐Ring) and a zwitterionic carboxy betaine (CB‐OH) to achieve the switch repeatedly (Figure 5B). The CB‐Ring surface could kill over 99.9% of *E. coli* K12 attached on it under dry conditions. In neutral or basic salt solutions, CB‐Ring was hydrolyzed to CB‐OH immediately. The CB‐OH surface released 90% adherent dead bacteria, resisted bacterial adhesion in the aqueous media, and prevented biofilm formation.

Commercially available poly(trimethylamino) ethyl methacrylate chloride (pTMAEMA) was modified by soaking with different electrolyte solutions (such as sulfate, citrate, hexametaphosphate) (Figure 5C).^[^ ^132^ ^]^ The adsorption of counter ions made the polymer surface transform from bactericidal to antibacterial adhesion, in the meanwhile released the adherent dead bacteria. The bactericidal rate of pTMAEMA surface reached at least 94.3% against *E. coli*, and released 98.3% attached *E. coli* along with the transformation. Finally, the surface could restore the bactericidal property by soaking in sodium chloride solution. Furthermore, Chen and co‐workers^[^ ^133^ ^]^ developed a poly(methacrylic acid) (PMAA) modified silicon nanowire arrays surface that could load and release lysozyme (Lys) in response to the solution pH by varying the kinds and concentrations of salts. The surface served as a self‐cleaning platform to kill and release bacteria on demand (Figure 5D). The killing efficiency of PMAA/Lys surface was more than 95%, and more than 90% of the previously attached bacteria could be released. Although salt‐responsive antibacterial strategy is easily affected by the environment, like pH of the solution, concentration of electrolytes, it is a convenient method to achieve switchable bacteria‐killing and bacteria‐releasing capabilities.

### 3.2 Design Principles of Bacterial Metabolites Stimuli‐Responsive Antibacterial Materials

Bacterial metabolites make up the microenvironment of infection. Bacterial metabolites stimuli can achieve real‐time self‐adaptive antibacterial activity, that detect and treat infections with respect to dosage, duration, and location. The real‐time antibacterial activity of bacterial metabolites responsive materials can prevent the following bacterial infection, biofilm formation, and the emergence of resistant bacteria efficiently. Acid‐responsive polymers contain acid‐responsive bonds, like acrylic polymers, Schiff base bond, and *β*‐carboxylic amide bond. Due to the specificity of enzyme, the structure of the enzyme‐responsive polymers corresponds to the kind of secreted enzymes. Redox‐responsive materials have the ability of redox reaction. The following sections explain these design principles in detail.

#### 3.2.1 Acid

Bacterial metabolism produces acids like acetic acid, lactic acid, malic acid, etc., which lower the pH of the microenvironment where bacteria‐infected (pH 4.5–6.5).^[^ ^134^, ^135^ ^]^ Based on this feature, the use of acid‐responsive polymers can control the release of bactericides (such as antibiotics, antibacterial peptides), and kill bacteria adaptively.^[^ ^135^ ^]^ This strategy can realize on‐demand bactericide release depending on the dosage, duration, and location of the infected bacteria.^[^ ^136^ ^]^ Avoiding the misuse of bactericides in response to pH of the microenvironment can reduce the emergence of resistant bacteria effectively. The commonly used acid‐responsive materials include: i) protonated polymers, such as polyacrylic acid (PAA), polymethacrylic acid (PMAA), TA, etc. to adsorb aminoglycoside antibiotics or antimicrobial peptides electrostatically; ii) acid‐responsive chemical bonds (Schiff base, dimethylmaleic anhydride, citraconic anhydride, etc.) to fix bactericides chemically.

**Figure 6:**
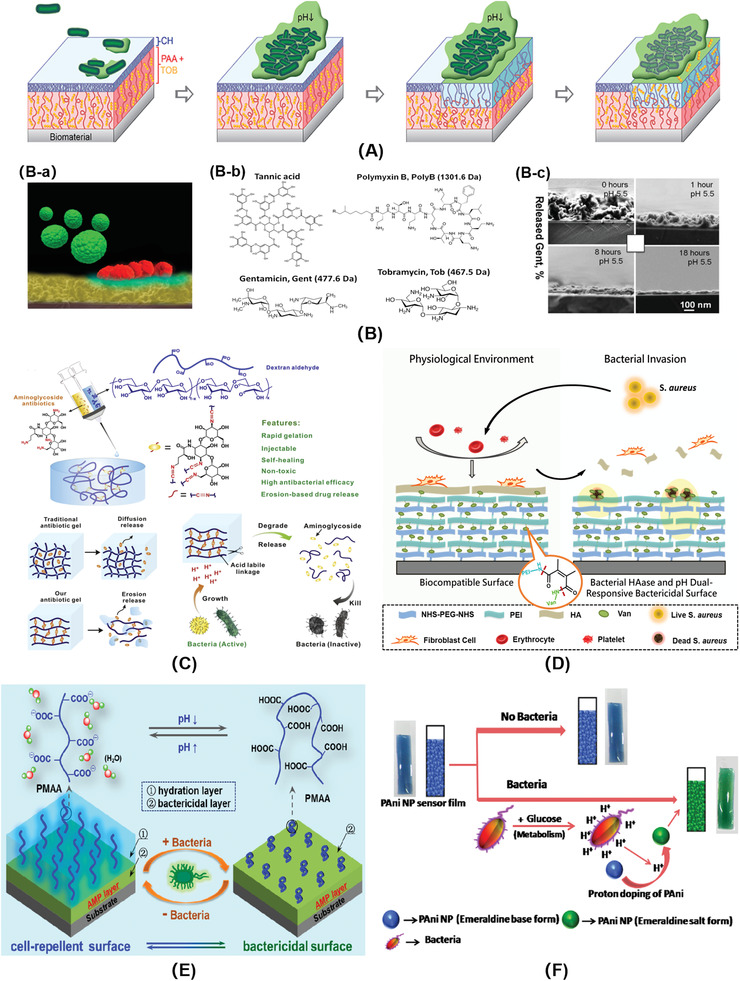
A) A pH‐responsive, drug release polymer bilayer system for the prevention of bacterial infection on biomaterials. Reproduced with permission.^[^ ^137^ ^]^ Copyright 2015, American Chemical Society. B‐a) Schematic diagram of bacterial infection microenvironment. B‐b) Chemical structures of polyelectrolyte multilayers components. B‐c) Cross‐sectional SEM images of a (TA/Gent)_300_ film at different release times. Reproduced with permission.^[^ ^138^ ^]^ Copyright 2014, American Chemical Society. C) The smart aminoglycoside hydrogels. The antibiotics served as linkers to form hydrogels with oxidized polysaccharides via an acid‐labile Schiff base linkage. Reproduced with permission.^[^ ^139^ ^]^ Copyright 2017, Elsevier. D) Schematic diagram of the bacterial responsive bactericidal and biocompatible surface.^[^ ^143^ ^]^ Copyright 2018, Royal Society of Chemistry. E) Nonleaching bacterial acid‐responsive antibacterial surface. Reproduced with permission.^[^ ^144^ ^]^ Copyright 2016, American Chemical Society. F) Schematic representation of the PAni‐Pec NPs based colorimetric sensor to monitor bacterial growth. Reproduced with permission.^[^ ^145^ ^]^ Copyright 2015, Elsevier.

Electrostatic interaction has been widely used to control antibiotics release. Lee et al.^[^ ^137^ ^]^ constructed an acid‐responsive bilayer surface consisting of inner layer of PAA brush and outer layer of CS brush (**Figure** **6**A). Under physiological condition, PAA was anionic, and aminoglycoside antibiotic tobramycin (Tob) could be attracted to PAA brush through electrostatic interactions. Meanwhile, CS chains were in a collapsed and dense state, which prevented the overflow of Tob and endowed the surface with better biocompatibility. When bacteria invaded, as the pH decreased, PAA became protonation and lost adsorption ability of Tob. Then, the outer layer of CS transferred into the cationic NH^3+^ state, making the molecular chains repel each other and stretch, which was suitable for the release of Tob and killed almost all the *S. aureus* colonies. Sukhishvili's group^[^ ^138^ ^]^ used TA to prepare bactericidal coatings through electrostatic interactions. Antibiotic aminoglycoside was loaded via layer‐by‐layer self‐assembly technology. The coating realized acid‐responsive release of antibiotics and eliminated 97% of *S. epidermidis* colonization (Figure 6B). However, loading bactericides through electrostatic attraction has the disadvantage of instability, which may be affected by many factors, such as solution ionic strength, pH, etc.^[^ ^64^ ^]^

Fixing bactericide via chemical reactions can realize more effectively controlled release and minimize the impact of the external environment. An aminoglycoside‐contained hydrogel was fabricated through acid‐labile Schiff base. Aminoglycosides were used as cross‐linking agents to integrate with aldehyde‐contained oxidized polysaccharides such as dextran, carboxymethyl cellulose, alginate, and chondroitin (Figure 6C).^[^ ^139^ ^]^ Acid generated by bacterial infection would break Schiff base linkage, disintegrate the hydrogel and speed up the release of antibiotics. The hydrogel modulus, degradation rate, and release kinetics could be precisely controlled by adjusting the content of aminoglycoside in the gelation. In addition, the prepared aminoglycoside hydrogel had advantages of rapid formation in situ, injectable, self‐healing, non‐cytotoxicity, on‐demand release of antibiotics, and excellent antibacterial activities.

Compounds with *β*‐carboxylic amide bearing materials (such as dimethyl maleic anhydride, citraconic anhydride) have acid‐responsive hydrolysis characteristics and have been widely used in anticancer drugs delivery by charge‐conversional mechanisms.^[^ ^140^, ^141^, ^142^ ^]^ The *β*‐carboxamide bond is stable at neutral pH, hydrolyzes slowly at pH 6, yet hydrolyzes immediately at pH 5, which matches the metabolic acidification process of bacteria. Luan and co‐workers^[^ ^143^ ^]^ constructed a vancomycin‐conjugated surface by layer‐by‐layer (LbL) assembly (Figure 6D). Antibiotic vancomycin (Van) was immobilized to the surface through acid‐labile *β*‐carboxamide linker, and was released with superior antimicrobial behavior in response to the lower pH because of bacterial infection.

Non‐leaching surfaces which kill bacteria on contact have long‐term antibacterial activities, and bactericides will not overflow to affect the surrounding environment. Furthermore, Luan's group^[^ ^144^ ^]^ developed a non‐leaching bacterial acid‐responsive antibacterial surface based on a unique hierarchical architecture. The poly(methacrylic acid) (PMAA) outer layer exhibited good biocompatibility and resisted the initial bacterial attachment under physiological conditions. Once the bacteria‐infected, the PMAA chains collapsed in response to the bacterial acidification, therefore exposing the underlying antimicrobial peptides to on‐demand kill bacteria. The killing efficiency changed from 9.3% at pH 7.4 to 77.5% at pH 5.0, indicating the pH‐activated antibacterial properties. Furthermore, the adherent dead bacteria could be released along with the stretching of the PMAA chains by increasing the environmental pH (Figure 6E). This report provides a new idea to avoid bacterial resistance without releasing bactericides.

The bacteria‐triggered acidification can also detect bacterial infection and monitor bacterial growth, which is important for disease prevention. PANI could change its color from blue to green which could be observed by naked eyes, and increase its conductivity to 8–10 orders of magnitude in response to bacterial acidification. This was attributed to its transition between emeraldine base (EB) form and emeraldine salt (ES) from, which was triggered by the bacterial acidic metabolites (Figure 6F).^[^ ^145^ ^]^ It could be seen at high inoculant cell density (≈10^8^ cells mL^−1^), the sensor showed significant change in pH within 5 min. Detection bacterial infection based on bacterial metabolites can realize real‐time monitoring of bacteria and reflect the degree of infection timely.

#### 3.2.2 Enzyme

Bacteria secrete a variety of enzymes during metabolism such as lipase, phosphatase, hyaluronidase, urease, gelatinase, coagulase, *β*‐lactamase, nitroreductase, etc.^[^ ^146^, ^147^, ^148^ ^]^ Enzyme‐catalyzed reactions have the advantages of high sensitivity, specificity and can be conducted under mild conditions.^[^ ^149^, ^150^ ^]^ Enzyme‐responsive bactericidal systems can release bactericide depending on the dosage, duration, and location of the infected bacteria, which provides a new method to improve the biocompatibility of bactericidal systems, control the release of drugs, and reduce the generation of drug‐resistant bacteria.^[^ ^80^, ^151^ ^]^

**Figure 7:**
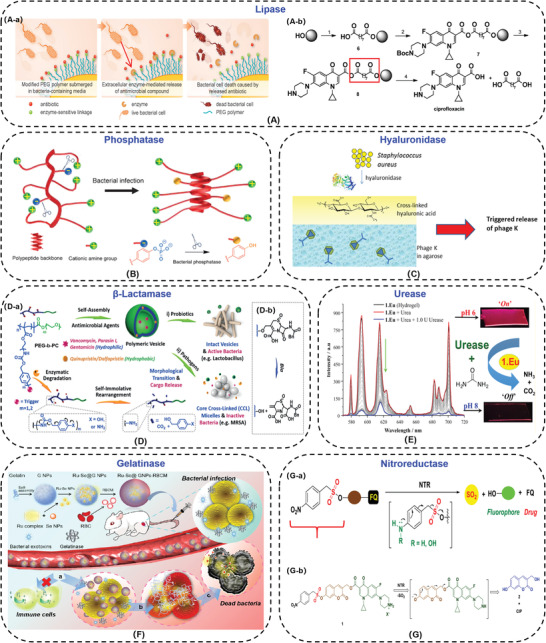
A‐a) Schematic of bacteria‐triggered enzymatic release of antibiotics from chemically modified polymers. A‐b) Solid‐phase synthesis of mixed anhydride 8 and bacterial lipase triggered release of ciprofloxacin. Reproduced with permission.^[^ ^154^ ^]^ Copyright 2014, Wiley‐VCH. B) Illustration of antimicrobial polypeptides with a random coil‐to‐helix conformation transition. Reproduced with permission.^[^ ^156^ ^]^ Copyright 2017, Wiley‐VCH. C) Schematic of bilayered hydrogel design. The lower layer contained ΦK particles and the upper layer comprised cross‐linked HA that is degraded by secreted bacterial HAase. Reproduced with permission.^[^ ^160^ ^]^ Copyright 2014, American Chemical Society. D‐a) Enzyme‐responsive polymeric vesicles for bacterial strain delivery of antibiotics selectively. D‐b) The changes of chemical structure induced by bacterial *β*‐lactamase (Bla). Reproduced with permission.^[^ ^162^ ^]^ Copyright 2016, Wiley‐VCH. E) Luminescent Lanthanide cyclen‐based enzymatic assay capable of diagnosing the onset of catheter‐associated urinary tract infections. Reproduced with permission.^[^ ^163^ ^]^ Copyright 2017, American Chemical Society. F) Illustration of the Ru‐Se@GNP‐RBCM nanosystem for detection and eradication of bacterial infection in response to gelatinase. Reproduced with permission.^[^ ^164^ ^]^ Copyright 2019, American Chemical Society. G‐a) The prodrug that is activated by nitroreductase (NTR) to generate an antibiotic and a fluorescent molecule. G‐b) The ciprofloxacin‐latent fluorophore conjugate 1, the fluorophore 2, and antibiotic ciprofloxacin (CIP). Reproduced with permission.^[^ ^165^ ^]^ Copyright 2019, American Chemical Society.

A majority of *S. aureus*, some *E. coli*, and *P. aeruginosa* have been proven to produce lipase, two *E. coli* strains Top10 and BL21 have low lipase secretions.^[^ ^152^, ^153^ ^]^ Komnatnyy et al.^[^ ^154^ ^]^ linked ciprofloxacin on the poly(ethylene glycol) (PEG) modified surfaces via anhydrides. In the physiological environment, the surface showed excellent biocompatibility. Once bacterial infection, the lipase was secreted by bacteria and ciprofloxacin was released to kill almost completely bacterial strains within 4 h at the early stage of biofilm formation. This self‐regulating system overcome the challenges of controlled release of antibiotics, bacterial resistance, and the formation of biofilm (**Figure** **7**A).

Phosphatase is another bacteria‐secreted enzyme, like *S. aureus*, *methicillin‐resistant S. aureus*, *Bacillus toyonensis*, and *Pseudomonas aeruginosa*, which hydrolyzes phosphorylated groups of responsive materials.^[^ ^155^, ^156^ ^]^ Xiong and co‐workers^[^ ^156^ ^]^ synthesized antimicrobial peptides with phosphorylated side chains, which was characterized by enzyme‐induced random‐coil to helix transition (Figure 7B). Anionic phosphorylated tyrosine was incorporated in the cationic antimicrobial peptides. Under normal physiological conditions, due to the electrostatic adsorption between negative‐charged and positive‐charged side chains, the antimicrobial peptides were in a random coil state, and exhibited low cytotoxicity. While at the *S. aureus* infected site, the phosphorylated groups of tyrosine were degraded by phosphatase, the helical structure of the antimicrobial peptide was restored, and recovered the bactericidal ability. The phosphatase‐responsive antimicrobial peptides activated the antibacterial activity selectively and reduced the non‐specific cytotoxicity against mammalian cells.

Hyaluronic acid (HA) has good biocompatibility which can promote the cell proliferation.^[^ ^157^ ^]^ Hyaluronidase (HAase) is secreted in the majority of Gram‐positive bacteria, such as *Staphylococcus*, *Streptococcus*, with little to no excretion in Gram‐negative strains, like *E. coli* DH5*α*.^[^ ^158^, ^159^ ^]^ Bean et al.^[^ ^160^ ^]^ utilized HA as a protective layer to construct bilayer antibacterial hydrogel. The lower layer of hydrogel was agarose containing antimicrobial virus Bacteriophage K (ΦK), and the upper layer was photo cross‐linked hyaluronic acid methacrylate hydrogel (Figure 7C). Under normal physiological conditions, cross‐linked HA endowed the surface better biocompatibility, and blocked the release of ΦK in the lower layer. In the presence of bacteria, the upper layer of HA was degraded by HAase, and ΦK was released to achieve bactericidal function. The killing ratio was more than 90%.

*β*‐Lactam antibiotics, like penicillins and cephalosporins, are widely used in hospital for treating bacterial infection. Unfortunately, a number of drug‐resistant bacterial strains, such as methicillin‐resistant *S. aureus* (MRSA), can secret *β*‐lactamases (Bla) to hydrolyze the *β*‐lactam ring and cause the resistance.^[^ ^148^, ^161^ ^]^ Fortunately, Liu's group^[^ ^162^ ^]^ utilized *β*‐lactamase responsive polymeric vesicles to deliver drug, that could treat drug‐resistant infections among probiotics selectively and avoid antibiotic misuse/abuse (Figure 7D). Polymeric vesicles self‐assembled from PEG‐b‐PC were subjected to side chain cleavage and microstructural transformation in response to Bla. Antibiotics were released to kill MRSA efficiently.

Urease‐producing microorganisms, specifically *Proteus mirabilis*, *Proteus vulgaris*, and *Providencia rettgeri*, most associated with catheter‐associated urinary tract infection.^[^ ^163^ ^]^ Urease hydrolyzes the urea in the urine into ammonia and carbon dioxide, causing the urine pH increased toward alkaline. Surender et al.^[^ ^163^ ^]^ constructed a supramolecular Eu(III)‐based catheter surface to indicate the invasion of *Proteus* by quenching the metal‐centered emission of 1.Eu (Figure 7E). This Eu(III)‐based macrocyclic probe (1 × 10^−5^ M) was able to signal both bacterial infection and biofilm formation in less than 60 min within catheters.

A variety of bacteria, like *S. aureus*, methicillin‐resistant *S. aureus*, *P. aeruginosa* have been proven to secrete gelatinase, thus gelatin could be used to deliver drugs.^[^ ^146^ ^]^ Liu and co‐workers^[^ ^164^ ^]^ constructed the bacteria‐responsive gelatin nanoparticles (25 µg mL^−1^) to detect and kill more than 90% methicillin‐resistant *S. aureus* (MRSA) by loading Ruthenium (Ru) and Selenium (Se) complexes into gelatin nanoparticles (Ru‐Se NPs) (Figure 7F). Gelatin could be degraded by gelatinase in situ, therefore, Ru‐Se NPs with intense fluorescence and antibacterial effects were released to detect and kill the infected MRSA.

Nitroreductase (NTR) is predominantly associated with *E. coli*.^[^ ^165^ ^]^ The ciprofloxacin‐latent fluorophore conjugate was synthesized. Upon *E. coli* infection, activated by NTR, the conjugate released ciprofloxacin (CIP) to kill the bacteria and the fluorescence signal was stronger to indicate bacterial infection (Figure 7G).^[^ ^165^ ^]^ The minimum inhibitory concentration (MIC) against *S. aureus* was 4 µg mL^−1^.

Enzyme‐responsive antibacterial materials have been used in antibiotics controlled release, preventing the formation of biofilms, reducing the production of drug‐resistant bacteria, avoiding the cytotoxicity of bactericides, specifically and sensitively detecting bacterial infection, and so on. They all associate with chemical structural transformations with good stability under the physiological environment. Furthermore, the ability to selectively kill drug‐resistant bacteria is very appealing. While the preparation of enzyme‐responsive antibacterial system is a little complicated. Simplifying the synthesis and preparation process is the future direction of enzyme‐responsive materials.

#### 3.2.3 Redox

**Figure 8:**
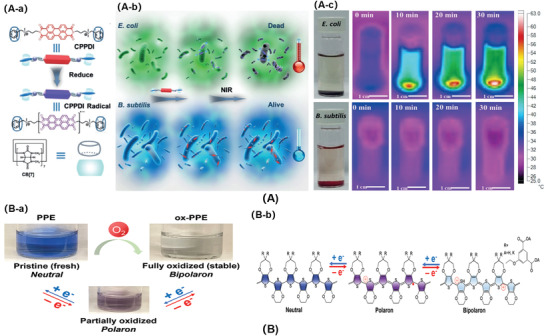
A‐a) Chemical structures of the designed supramolecular complex (CPPDI) and CPPDI radical anions. A‐b) Diagram of CPPDI selectively killing *E. coli* over *B. subtilis* in response to reducing agents. A‐c) Temperature changes of the aqueous solution of CPPDI in the presence of *E. coli* and *B. subtilis* under NIR irradiation. Reproduced with permission.^[^ ^166^ ^]^ Copyright 2017, Wiley‐VCH. B‐a) Schematic illustration of PPE in different oxidation states. B‐b) Photographic images of PPE aqueous solutions in neutral state (with N_2_H_4_ reducing agent), or in polaron/bipolaron (ox‐PPE) state via ambient oxygen oxidation. Reproduced with permission.^[^ ^88^ ^]^ Copyright 2020, Wiley‐VCH.

Bacteria produce reducing secretions like cysteine, glutathione (GSH), and thiol proteins into the medium. Those metabolites provide another possible mechanism for bacterial detection and killing. Traditional photothermal sterilization relies on non‐specific interactions, which have no bacterial selectivity. Zhang and co‐workers^[^ ^166^ ^]^ designed a new type of redox responsive supramolecular complex to selectively inhibit facultative anaerobes through PTT (**Figure** **8**A). First, a water‐soluble monomer of perylene diimide derivative was synthesized, and coordinated with cucurbit[7]uril to prepare supramolecular complex (CPPDI) through host–guest interactions. Facultative anaerobic bacteria such as *E. coli* had ability to reduce CPPDI to CPPDI radical anions, while aerobic bacteria such as *B. subtilis* did not have sufficient reducing ability to induce CPPDI radical anions in situ. The CPPDI radical anions could convert light energy into thermal energy and the temperature was risen up to 65 ℃ in 30 min, that exhibited excellent photothermal antibacterial effect (≈99%) (Figure 8A(c)). The redox responsive PTT was expected to regulate microbial balance by selective activation.

The reduction reaction is noticeably different among various bacterial strains. Redox‐responsive materials can be used to distinguish Gram‐negative and Gram‐positive bacteria, due to the different metabolites. The aqueous soluble oxidized electrochromic polymer ox‐PPE (poly(3,4‐propylenedioxythiophen‐alt‐3,4‐ethylenedioxythiophene) copolymer) was prepared. Based on the property of bacterial trans‐plasma membrane electron transport, ox‐PPE showed a visual color change in response to reducing agents secreted by live bacteria (Figure 8B). The Gram‐positive bacteria was clearly distinguished from susceptible Gram‐negative bacteria by using ox‐PPE within 30 min, because most Gram‐positive bacteria did not produce GSH, while other alternative thiols had less negative redox potential than GSH. Besides, ox‐PPE could probe the bacterial susceptibility to different antibiotics, owning to the decrease of the metabolic activity.^[^ ^88^ ^]^

The redox‐responsive materials can discern different reduction products secreted by different bacterial strains. They can detect or kill bacteria selectively and specifically. The specific bactericidal ability among various bacterial strains is charming for antibacterial treatment.

### 3.3 Design Principles of Multiple Stimuli‐Responsive Antibacterial Materials

Multiple stimuli‐responsive antibacterial systems can target the infected location and detect the bacterial invasion timely in response to bacterial metabolism products, thus improving the bactericidal effect and biocompatibility.^[^ ^167^ ^]^ Multiple stimuli‐responsive antibacterial systems regulate smarter, possess multi‐functions, and can kill the drug‐resistant bacteria, bacteria in biofilms, and prevent the formation of biofilms. Multiple stimuli can be divided into the combination of physical stimuli and physical stimuli, the combination of bacterial metabolites stimuli and physical stimuli, the combination of bacterial metabolites stimuli and bacterial metabolites stimuli.

**Figure 9:**
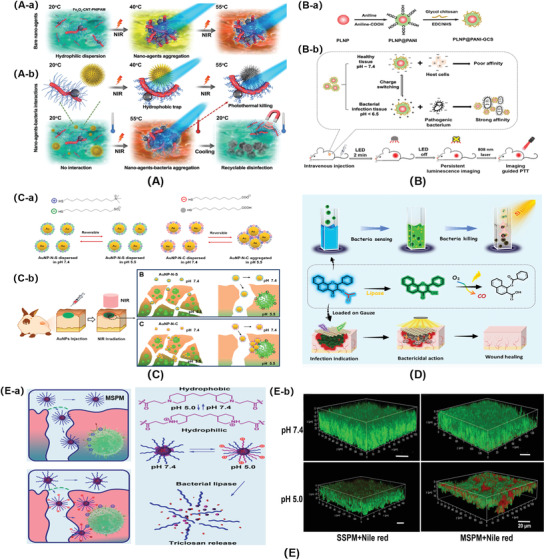
A‐a) The nano‐agents that transferred to hydrophobic and form robust aggregations once the temperature above 40 °C. A‐b) The hydrophobic trapping and photothermal killing progress of the nano‐agents. Reproduced with permission.^[^ ^168^ ^]^ Copyright 2018, Wiley‐VCH. B‐a) Schematic for the preparation of PLNP@PANI‐GCS. B‐b) Illustration of PLNP@PANI‐GCS for persistent luminescence imaging‐guided PTT of bacterial infection. Reproduced with permission.^[^ ^170^ ^]^ Copyright 2020, Wiley‐VCH. C‐a) Schematic illustration of the pH stimulus of AuNPs. C‐b) Effect of an acidic trigger approach onto the effective adsorption and enhanced photothermal ablation of MRSA biofilm. Reproduced with permission.^[^ ^171^ ^]^ Copyright 2017, American Chemical Society. D) Schematic concept of bacterial sensing of the CORM‐Ac process and subsequent bacterial killing in situ. Reproduced with permission.^[^ ^172^ ^]^ Copyright 2020, Royal Society of Chemistry. E‐a) The targeting and hydrolysis of mixed‐shell polymeric micelles (MSPMs) under the influence of bacterial pH changes and lipase degradation. E‐b) CLSM micrographs demonstrating penetration and accumulation of Nile red loaded MSPMs into the biofilm of *S. aureus* at pH 5.0. Reproduced with permission.^[^ ^76^ ^]^ Copyright 2016, American Chemical Society.

Photothermal conversion antimicrobial materials combined with thermo‐responsive materials, and magnetic materials, could effectively capture, kill and release bacteria recyclability (**Figure** **9**A).^[^ ^168^ ^]^ PNIPAM polymer brushes were grafted onto carbon nanotube‐Fe_3_O_4_. Due to the temperature sensitivity of PNIPAM, nanoparticles could be transferred from hydrophilic dispersions to hydrophobic aggregates under NIR irradiation. The formed aggregates were used as local heat sources to enhance the photothermal antimicrobial effect. The killing ratio was nearly 100% to both *S. aureus* and *E. coli* after minutes of irradiation. After cooling, more than 95% of dead bacteria could be released, and the magnetic nanoparticles were recovered and reused for recyclable disinfection capability.

Photothermal conversion antimicrobial materials combined with acid‐responsive materials could target the bacterial acidic microenvironment and kill resistant bacteria efficiently. Self‐doped PANI micelles were reported to kill bacteria through PTT. The CS derivative containing PANI in side chains could self‐assemble to form nano‐scale micelles in the aqueous environment.^[^ ^169^ ^]^ When touched the marginal healthy tissue (pH 7.0–7.4), the colloidal gel was formed to prevent the micelles from spilling onto healthy tissues. Once the bacteria‐infected, the nano‐sized micelles were not charged in the acidic bacterial infection area (pH 6.0–6.6) and spread over the acidic abscesses, since the pKa of CS was 6.0–6.5. Under NIR irradiation for 20 min (808 nm, 2.0 W cm^−2^), PANI could absorb light energy and convert it into thermal energy, the temperature of the infected area increased to kill bacteria and the killing ratio was more than 96%. Recently, PANI has been demonstrated that it owned a higher light‐heat conversion property at pH 6.5 than pH 7.4. Based on this property, Yan et al.^[^ ^170^ ^]^ constructed a pH switchable antibacterial nanoplatform PLNP@PANI‐GCS, by grafting PANI and glycol CS (GCS) onto the surface of persistent luminescence nanoparticles (PLNPs) (Figure 9B). PLNP@PANI‐GCS took the advantage of the long persistent luminescence of PLNPs to image bacterial infection and realized the accurate PTT in response to bacterial acid microenvironment. The PLNP@PANI‐GCS killed over 99% of the three kinds of bacteria (*E. coli*, *S. aureus*, and methicillin‐resistant *S. aureus*) under NIR laser irradiation for 8 min (808 nm, 1.5 W cm^−2^). Gold nanoparticles (AuNPs) modified with (10‐mercaptodecyl)trimethylammonium bromide and 11‐mercaptoundecanoic acid mixed self‐assembled monolayers (AuNP‐N‐C), could target the acidic microenvironment of bacterial infection and photothermal ablation of methicillin‐resistant *S. aureus* (MRSA) biofilm. In MRSA biofilm, the acidic condition prompted AuNP‐N‐C to possess positive charge and effectively adsorbed to bacteria with negative charge. By targeting effect, the AuNP‐N‐C exhibited great photothermal bactericidal property under NIR light irradiation without damage to surrounding healthy tissues (Figure 9C).^[^ ^171^ ^]^ The temperature increased rapidly in the first 2 min and showed a maximum temperature of approximately 60 °C. The MRSA biofilm presented obvious structural destruction, and most of the bacteria were killed.

Bacterial enzyme‐responsive materials combined with photo‐induced gas release materials could obtain bacterial infection theranostics. Photoactivatable organic carbon monoxide releasing molecule (CORM‐Ac) was synthesized, and could detect and eliminate bacterial infection successfully in response to bacterial lipase and light.^[^ ^172^ ^]^ As illustrated in Figure 9D, CORM‐Ac gave an early warning on infection via the visualized fluorescence signal by cleavage of the O‐acetyl group triggered by bacterial lipases. Then CO was released from CORM to kill the invading bacteria upon a simulated sunlight source. This “sense‐and‐treat” CORM‐Ac molecular represented a potential strategy for precise infection treatment. The CO showed significant bactericidal abilities on methicillin‐resistant *S. aureus* and ≈62% biofilm biomass was moved after CORM‐Ac/light treatment.

Dual bacterial metabolites stimuli can complete bactericidal process without any outside intervention. Henny C. van der Mei^[^ ^76^ ^]^ prepared the poly(*β*‐amino ester) nanomicelles with adaptive surface charges to load antibiotic triclosan and killed bacteria in biofilms (Figure 9E). The mixed core‐shell polymer micelles consisted of hydrophilic PEG in shell and acid‐responsive poly(*β*‐amino ester) in core (MSPMs). The mixed micelles were negatively charged under the physiological environment, which facilitates the penetration into biofilm. Under low pH condition of bacterial surfaces, the micelles were positively charged, and electrostatically targeted the negatively charged bacteria. Furthermore, the ester groups of micellar polymer chains were hydrolyzed by bacterial lipase, thereby releasing the encapsulated triclosan and inactivating bacteria in the biofilm efficiently at a low encapsulated triclosan concentration of around 5 µg mL^−1^. As a comparison, the single‐shell polymeric micelles with only PEG‐shell (SSMPs) could not penetrate into the biofilm regardless of the pH (Figure 9E(b)).

Multiple stimuli‐responsive antibacterial systems apply various responsive materials to achieve all kinds of required antibacterial functions on demand flexibly, including intractable drug‐resistant bacterial infections and bacterial biofilm infections. Dual bacterial metabolites stimuli can indicate infected location precisely and control bactericidal property without any human interventions.

## 4 Biomedical Applications

Smart stimuli‐responsive antibacterial materials can be applied in the field of medical devices, drug delivery, theranostics, tissue engineering, and so on.^[^ ^147^, ^173^, ^174^, ^175^, ^176^ ^]^ These applications can be achieved by surface coating, hydrogel, electrospinning, spray, intravenous injection, etc. We discuss the specific explanations and examples in the following sections.

### 4.1 Medical Devices

**Figure 10:**
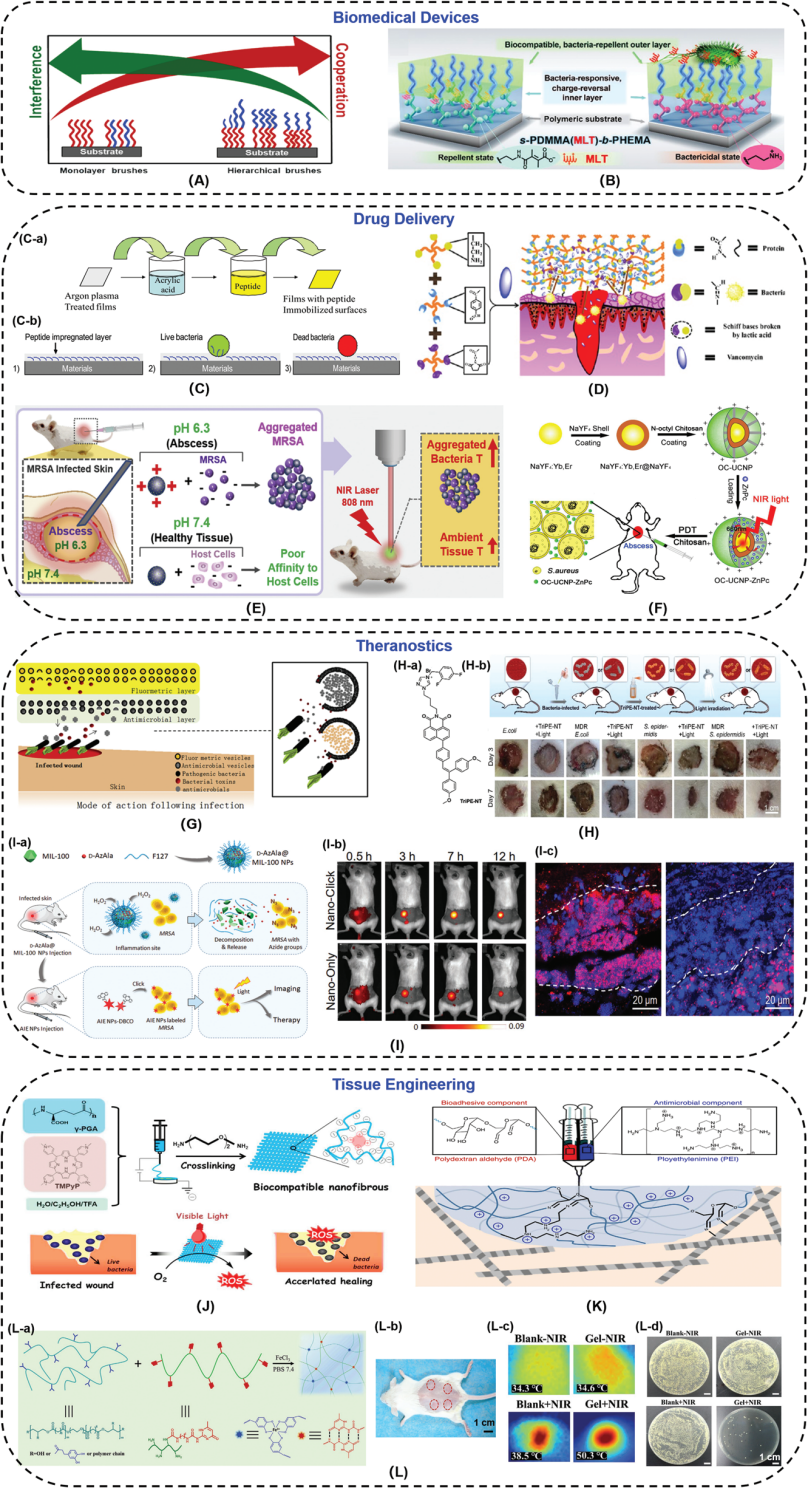
A) The monolayer polymer brushes and the hierarchical polymer brushes. Reproduced with permission.^[^ ^184^ ^]^ Copyright 2021, Elsevier. B) Schematic diagram of the antibacterial mechanism of the hierarchical platform. Reproduced with permission.^[^ ^186^ ^]^ Copyright 2020, American Chemical Society. C‐a) Schematic of lytic peptide immobilization on indwelling device surfaces. C‐b) Illustration of the acting mechanism of bacterial responsive antibacterial surfaces. Reproduced with permission.^[^ ^67^ ^]^ Copyright 2015, Elsevier. D) Vancomycin‐loaded hydrogel system, and its bacterial responsive release of vancomycin. Reproduced with permission.^[^ ^192^ ^]^ Copyright 2016, American Chemical Society. E) The wound treated with PANI‐GCS NPs via subcutaneous injection and killing bacteria by acidity triggered aggregation and photothermal ablation. Reproduced with permission.^[^ ^193^ ^]^ Copyright 2017, Elsevier. F) Schematic diagram of OC‐UCNP‐ZnPc synthesis and its antibacterial activity in vivo. Reproduced with permission.^[^ ^194^ ^]^ Copyright 2017, Royal Society of Chemistry. G) Schematic of intelligent wound dressing construction and the mode of action following infection. Reproduced with permission.^[^ ^198^ ^]^ Copyright 2018, Elsevier. H‐a) The chemical structure of TriPE‐NT. H‐b) In vivo evaluation of TriPE‐NT in treatment of bacterially infected wounds on rats by spray. Reproduced with permission.^[^ ^200^ ^]^ Copyright 2018, Wiley‐VCH. I‐a) Schematic illustration of the proposed strategy of bacterial diagnosis and therapy by the H_2_O_2_‐responsive MOFs assisted in vivo metabolic labeling of bacteria. I‐(b) Time‐dependent in vivo fluorescence images of bacteria‐bearing mice pretreated with d‐AzAla@MIL‐100 NPs (Nano‐Click group) or saline (Nano‐Only group), respectively, followed by injection of AIE NPs. I‐c) Fluorescence images of the infected skin slices of mice. The areas within the dotted lines indicated bacterium aggregation on the infected skin. Reproduced with permission.^[^ ^201^ ^]^ Copyright 2018, Wiley‐VCH. J) Schematic illustration of fabrication of poly(*γ*‐glutamic acid)/TMPyP nanofibrous mat with PDT antibacterial activity and its application as wound dressing. Reproduced with permission.^[^ ^205^ ^]^ Copyright 2020, Elsevier. K) Bioadhesive hydrogels with antibacterial activity. Reproduced with permission.^[^ ^207^ ^]^ Copyright 2014, Nature Publishing Group. L‐a) Fabrication of PEGSD/GTU hydrogel. L‐b) Photograph of mouse with subcutaneous abscesses before treatment. L‐c) The thermographic images of subcutaneous abscess after treatment. L‐d) Photographs of the survival MRSA clones on the agar plates from the infected tissues of mice treated with different experimental conditions. Reproduced with permission.^[^ ^208^ ^]^ Copyright 2020, Wiley‐VCH.

Stimuli‐responsive materials can be loaded onto the medical device surfaces via surface coating. Surface coating is a simple method to obtain antibacterial function and has been widely used in wound dressing, catheter, and other medical devices.^[^ ^177^, ^178^, ^179^ ^]^ Antibacterial coatings constructed with stimuli‐responsive polymers can kill bacteria timely, resist the formation of bacterial biofilms effectively, and reduce the generation of resistant bacteria.^[^ ^180^, ^181^, ^182^ ^]^ Antibacterial strategy involved repelling bacterial adhesion and killing bacteria. A large number of polymer surfaces integrated antifouling and bactericidal function into a single platform, that compromised one performance due to the random arrangement and spatial interference of different functional units.^[^ ^183^ ^]^ Luan's group^[^ ^184^, ^185^ ^]^ focused on constructing hierarchical polymer brushes on surface by grafting inner polymer layer onto the outer layer via living graft polymerization, that could control the drug delivery and minimize the interference between different grafted segments (**Figure** **10**A). The cationic antimicrobial peptide could be loaded into the anionic inner layer via electrostatic attraction. Under the physiological environment, the hierarchical polymer brushes on surface could inhibit the early bacterial adhesion and biofilm formation. Moreover, the hierarchical surface shielded the toxicity of the underlying antimicrobial peptide with the help of the hydrophilic upper layer. Once the bacteria‐infected, the acid microenvironment triggered the cleavage of labile amide bonds to release antimicrobial peptide allowing the surface to kill the adherent bacteria timely, and the bactericidal efficiency was more than 90% (Figure 10B).^[^ ^186^ ^]^ Stimuli‐responsive materials on medical device surfaces realize detection bacterial infection timely, and the bactericidal performance on demand with good biocompatibility and no biofilm formation.

### 4.2 Drug Delivery

Stimuli‐responsive antibacterial materials are widely used in drug delivery, that can release drug on demand and reduce the generation of drug‐resistant bacteria. Drugs can be loaded in polymer brushes on various surfaces, in hydrogels and subcutaneous injection solution.^[^ ^187^, ^188^ ^]^ Traba and Liang^[^ ^67^ ^]^ constructed bacterial responsive surfaces by lytic peptides and poly(acrylic acid) via layer by layer (Figure 10C). Once bacterial adhesion, the modified surface could release lytic peptides at the contamination sites in response to bacterial acidic microenvironment and prevent biofilm formation for up to a week on indwelling device efficiently. This method expanded the service life of indwelling device and alleviated the suffering of patients.

Hydrogels are very suitable for drug delivery, that can load drugs conveniently and release in a controlled manner in response to multi‐stimuli.^[^ ^189^, ^190^, ^191^ ^]^ Vancomycin (Van) was incorporated in the hydrogel which was formed by Schiff‐base reaction of different contents of 4‐arm‐PEG‐NH_2_, 4‐arm‐PEG‐NHS, and 4‐arm‐PEG‐CHO. Once infected, the cross‐links were hydrolyzed, Van was released in response to the degree of acidification, and almost all bacteria were killed when the concentration of vancomycin was 250 µg mL^−1^ in hydrogel (Figure 10D).^[^ ^192^ ^]^

Subcutaneous injection is widely used to deliver nanodrugs and the targeting effect could be achieved easily without blood circulation. The PANI conjugated glycol CS nanoparticles (PANI‐GCS NPs) were injected subcutaneously into the abscess. PANI‐GCS NPs were bacterium‐specific aggregated by surface charge conversion in response to bacterial acidification. Upon NIR irradiation, PANI converted light energy into thermal energy to completely ablate methicillin‐resistant *S. aureus* effectively (Figure 10E).^[^ ^193^ ^]^ A core‐shell upconversion nanoparticle (OC‐UCNP‐ZnPc), which was coated with cationic *N*‐octyl CS and loaded the PS zinc phthalocyanine, was also injected subcutaneously into the infected wound. The nanoparticle with high bactericidal efficiency, could kill drug‐resistant bacteria in deep tissue (1 cm) through the dual bactericidal activities of cationic *N*‐octyl CS PDT‐induced ROS (Figure 10F).^[^ ^194^ ^]^

Controlled drug release is indivisible with stimuli‐responsive materials. How to combine the responsive groups and the carriers appropriately to obtain controlled release is the focus of research. Electrostatic interaction, Schiff base, *β*‐carboxamide bond combined with surface coating, hydrogel, and subcutaneous injection are the commonly used methods. However, these methods are not via blood circulation. How to realize intravenous injection or oral drugs which release drugs after blood circulation is the future research direction.

### 4.3 Theranostics

Theranostics integrates accurate diagnosis and therapeutic capacities into a single system, that can generate real‐time diagnostic signals and facilitate the in situ therapeutic processes in time. Nowadays, pathogen diagnosis and therapy are independent processes in the conventional approach, inevitably leading to compromised therapeutic efficacy and increased economic and mental burdens. Sensitive bacterial detection facilitates the timely interventions at the early stage of infection before biofilm formation. The formation of biofilm can be divided into five stages: initial attachment of bacterial cells to the surface; irreversible adhesion stage; early development of biofilm architecture; maturation of biofilm architecture; dispersion of single cells from the biofilm.^[^ ^195^, ^196^ ^]^ It is well known that exist in mature biofilm, bacterial cells can become 100–1000 times more resistant to antimicrobial agents than planktonic bacteria.^[^ ^197^ ^]^ Detection and killing bacteria at the early stage of infection can avoid the formation of mature biofilm, inhibit the emergence of drug‐resistant bacteria, and reduce the difficulty of treatment efficiently.

Fan's group^[^ ^198^ ^]^ developed a theranostic surface composed of a bactericidal material in lower layer and a detection material in upper layer (Figure 10G). When infection occurred, the toxins or enzymatic factors secreted by pathogenic bacteria (e.g., methicillin‐resistant *S. aureus* and *P. aeruginosa*) would damage the vesicles to release the self‐quenching dye and antimicrobials to indicate the bacterial infection and inhibit the bacterial growth completely. The “sense‐and‐treat” surface could accelerate wound healing and reduce the difficulty of treatment via preventing bacterial growth and biofilm formation at the early stage of infection.

Spray is a simple and convenient method to deal with wound infection to achieve theranostic.^[^ ^199^ ^]^ Tang and co‐workers^[^ ^200^ ^]^ synthesized a bifunctional theranostic molecule, triphenylethylene‐naphthalimide triazole (TriPE‐NT), which could monitor and kill multidrug‐resistant bacteria through simple spay treatment (Figure 10H). The electrostatic attraction enabled the aggregation of TriPE‐NT around bacteria to induce aggregation‐induced emission luminogen (AIEgen) effect to monitor bacteria. Under the illumination of white light (4 mW cm^−2^, 30 min), TriPE‐NT generated ROS and could kill all *E. coli*, more than 96% multidrug‐resistant *E. coli*, 98% *S. aureus*, and more than 97% multidrug‐resistant *S. aureus*. The sense and treat process was achieved by spaying TriPE‐NT solution and imposing external stimuli easily.

Intravenous injection is a common medical approach. The challenge to intravenous treatment achieving theranostic is obtaining specific metabolic labeling in vivo. Liu's group^[^ ^201^ ^]^ utilized MIL‐100 (Fe) nanoparticles (NPs) to deliver target molecule and achieve theranostic in vivo. MIL‐100 (Fe) NPs released the target molecule 3‐azido‐D‐alanine to selectively mark bacteria in response to H_2_O_2_ which is secreted by tissues at the inflammation site. Then, PS NPs with aggregation‐induced emission characteristics were injected to react with marked bacteria through in vivo click chemistry. The aggregation of PS NPs around the bacteria‐induced the fluorescence signals, and the bactericidal effect was obtained through PDT (Figure 10I). The bacterial killing efficiency of the NPs reaches more than 75% under white light (300 mW cm^−2^) for 10 min. These MIL‐100 (Fe) NPs assisted system achieved detection and killing bacteria in vivo and prevented the infection from developing and spreading before the biofilm formation by intravenous injection.

Theranostics based on bacterial metabolites stimuli‐responsive materials can achieve detection and infection treatment without complicated operation. The application of stimuli‐responsive materials in theranostics has greatly promoted the development of intelligent medicine. The future direction of bacterial theranostics is to realize specific recognition and killing in vivo.

### 4.4 Tissue Engineering

Stimuli‐responsive materials are applied in tissue engineering via the forms of electrospinning, hydrogel, and so on.^[^ ^202^ ^]^ Electrospinning with high surface area, adjustable porosity, alignment, and stacking, has been used in a variety of biomedical applications, including tissue engineering scaffolds.^[^ ^203^, ^204^ ^]^ Fabricating electrospun nanofibers with PS can achieve PDT to resist bacterial infection. A *γ*‐PGA‐based nanofibers with PS were fabricated by co‐electrospinning of polyanionic *γ*‐PGA and cationic PS 5,10,15,20‐tetrakis(1‐methylpyridinium‐4‐yl)porphyrin tetra (p‐toluenesulfonate) (TMPyP) with different contents (Figure 10J).^[^ ^205^ ^]^ Due to the high solubility and electrostatic interactions, TMPyP could be well‐dispersed in the nanofibers without self‐quenching. This nanofibrous mat killed almost all bacteria efficiently upon 60 min light irradiation (650 ± 10 nm, 3 mW cm^−2^) and accelerated the healing process of wounds.

Hydrogel has been widely used in tissue engineering, relying on the large water content and flexibility.^[^ ^176^, ^206^ ^]^ Hydrogels with innate antibacterial polymers can realize stimuli‐responsive bactericidal property without drug release. Giano et al.^[^ ^207^ ^]^ developed the acid‐responsive branched polyethylenimine (PEI) hydrogels by injecting polydextran aldehyde and branched polyethylenimine in situ via Schiff‐base reaction (Figure 10K). The bacterial acid microenvironment induced Schiff‐base links hydrolysis, and the amino‐groups of branched polyethylenimine were positively charged to kill bacteria. The minimum inhibitory concentration (MIC) of soluble branched PEI was 0.09 mg mL^−1^ against *E. coli* and 0.1 mg mL^−1^ against *S. aureus*. The light‐responsive hydrogels were obtained by simply mixing the solutions of FeCl_3_, poly(glycerol sebacate)‐co‐poly(ethylene glycol)‐g‐catechol prepolymer (PEGSD), and ureido‐pyrimidinone‐hexamethylene diisocyanate synthon modified gelatin (GTU) (Figure 10L).^[^ ^208^ ^]^ After 3 min NIR irradiation (808 nm, 1.4 W cm^−2^), the catechol‐Fe^3+^ coordination could absorb the NIR and the temperature increased to 50 °C. After 10 min irradiation, the hydrogel groups showed almost 100% killing ratios against both *E. coli* and *S. aureus*, and 99.3% killing ratio against methicillin‐resistant *S. aureus* (MRSA).

Physical responsive materials like thermo‐sensitive PNIPAM, light‐sensitive PANI, and bacterial metabolites responsive materials like polyacrylic acid, CS, Schiff base, hyaluronic acid, etc. have been widely used in medical devices, drug delivery, theranostics, and tissue engineering. Stimuli‐responsive antibacterial materials can realize intelligent and personalized medicine with good biocompatibility and no drug‐resistance.

## 5 Conclusions and Perspectives

This article summarizes the research of smart stimuli‐responsive antibacterial materials in recent years systematically and comprehensively. The main issues with bacterial infections are the rapid build‐up of quasi‐impermeable biofilm and the resistance to different types of interventions. Compared with traditional antibacterial materials, smart stimuli‐responsive antibacterial materials can detect infections timely, activate bactericidal activity on‐demand, avoid bacterial resistance, prevent the formation of bacterial biofilms, kill the bacteria in biofilms, and alleviate cytotoxicity of bactericides effectively.

In order to solve the problem of the rapid build‐up of biofilm, first, the antifouling surface composed of thermo‐responsive materials and salt‐responsive materials are constructed. The antifouling surface can resist the initial adhesion of bacteria, thereby interfering with the earliest stages of biofilm formation. The antifouling strategy has been widely used in medical devices to deal with bacterial infection and biofilm formation. Second, bacterial metabolites stimuli‐responsive materials that achieve real‐time self‐adaptive antibacterial activity, can prevent the following bacterial infection and biofilm formation timely at the early stage. Bacterial resistance is a very tricky problem. Excitingly, light‐responsive materials including materials for PDT, PTT, and photo‐induced gas therapy, do not induce drug‐resistance and can kill drug‐resistant bacteria, relying on their bactericidal mechanisms. Second, thermo‐responsive materials can reduce the exposure time of antibiotics in response to temperature difference between body's internal and external environment. Third, bacterial metabolites stimuli‐responsive materials that release or expose antibiotics on demand, can treat the infection timely while avoiding the misuse of antibiotics, thus reducing the emergence of resistant bacteria. Especially, enzyme‐responsive antibacterial materials have the ability to selectively kill drug‐resistant bacteria. Stimuli‐responsive antibacterial materials have been widely used in drug delivery area to solve the problem of bacterial resistance. Furthermore, some multiple stimuli‐responsive antibacterial materials have achieved killing resistant bacteria in biofilm without inducing drug‐resistance. Theranostic that combines detection and killing capability, can avoid the formation of mature biofilm and inhibit the emergence of drug‐resistant bacteria efficiently.

The stimuli‐responsive antibacterial materials can be divided into materials that respond to physical stimuli and materials that respond to bacterial metabolites stimuli. The physical responsive antibacterial materials, with strong controllability, are convenient to operate, such as temperature, light, electricity, salt, and so on. The physical responsive materials are mostly designed by adjusting the entire molecular structure. For example, PEG analogs have been regulated the proportion and chain length of each component to obtain the transition between body temperature and room temperature. The bacterial metabolites stimuli‐responsive antibacterial materials can adaptively kill bacteria on demand according to the location and dosage of infection without any human interventions, such as acid, enzyme, redox, etc. The bacterial metabolites responsive materials achieve response characteristics via specific chemical bonds, like acid and enzyme sensitive bonds. The bacterial metabolites responsive materials, especially enzyme‐responsive materials, have the potential to achieve specific and selective sterilization. Multiple stimuli‐responsive antibacterial systems can design to integrate various responsive materials on demand, and achieve all kinds of required antibacterial functions flexibly. Both the physical responsive antibacterial materials and the bacterial metabolites responsive antibacterial materials can treat the infection timely, inhibit the formation of bacterial biofilm at the early stage, and reduce the generation of resistant bacteria efficiently. The responsive materials have a wide range of biomedical applications, including medical devices, drug delivery, theranostics, tissue engineering. Stimuli‐responsive antibacterial materials are appealing to realize intelligent and personalized medicine.

Although a lot of research has been made in stimuli‐responsive antibacterial materials, new strategies can be studied as follows: First, the incidence of multi‐resistant bacterial infection continuously increases, it is urgent to develop new bactericidal drugs and responsive systems to avoid the abuse of antibiotics. Once the invasion occurs, bacteria will rapidly proliferate to form biofilms, and increase the resistance to host immune cells and bactericides.^[^ ^209^ ^]^ Theranostic that combines real‐time detection and killing capability, can avoid bacterial reproduction and biofilm formation at the early stage of infection, thus becoming future research focus to deal with antibiotic resistance. Second, designing antibacterial materials, that can selectively kill pathogens based on the type of infected bacteria and protect probiotics and mammalian cells, is necessary to maintain the normal physiological environment of the human body. In the end, majority of reported response systems are still in the conceptual and model stage, it is necessary to develop simple, efficient, and universal responsive antibacterial materials, and apply these materials to clinical applications.

## Conflict of Interest

The authors declare no conflict of interest.
